# 1016. Case Study. Recognizing Likely Malignant Brain Edema in Severe Burn Injury

**DOI:** 10.1093/jbcr/irag033.284

**Published:** 2026-04-06

**Authors:** Richard B Arbour, Katie M Mapula

**Affiliations:** Temple University Hospital/Temple University Health System, Philadelphia, PA; Parkland Health, Keller, TX

## Abstract

**Patient Presentation (age range, injury details, relevant history):**

We present the case of a 30-50 year-old patient following large (greater than 65%) TBSA flame burn from a house fire. Patient had no significant past medical history and was responding appropriately on initial assessment by pre-hospital providers. Burns were circumferential around chest and abdomen.

**Clinical Challenges:**

Patient required advanced airway management, controlled ventilation and large-volume resuscitation guided by invasive pressure monitoring. Elevated airway pressures and patient-ventilator dys-synchrony necessitated central escharotomies to facilitate ventilation and mitigate intra-abdominal hypertension. Balancing resuscitation needs with risk of widespread tissue edema in non-burned areas was difficult.

**Management Approach:**

Large volume crystalloid, colloid and blood administration managed relative/absolute hypovolemia and significant intravascular volume loss to interstitial tissue. Central escharotomies were performed as clinically appropriate. On ICU day-4, a temporal relationship was noted among a flaccid neurological evaluation, loss of brainstem reflexes and marked cardiovascular instability becoming vasopressor-dependent.

**Outcomes:**

Blood pressure was supported with escalating vasopressors dosing and volume management. Family communication was maintained related to goals of care and expected mortality. On ICU day-5 family elected to transition to comfort-directed care and natural death.

**Lessons Learned:**

Large TBSA burns cause vasodilatation, inflammation, vascular endotheliopathy and widespread edema even in non-burned body areas. A temporal relationship among marked cardiovascular instability becoming vasopressor-dependent, flaccid neurological examination and loss of all brainstem reflexes may indicate terminal intracranial compartment syndrome with brainstem herniation. When occurring in the context of large-volume resuscitation and large TBSA burn injury, this may be a clinical trigger for formal brain death testing and inform family discussion around expected mortality and directions of care.

**Applicability to Practice:**

Close surveillance of neurological status including relationship among loss of neurologic function and brainstem reflexed and cardiovascular instability may indicate terminal brain herniation. When patients are encountered as described, these findings can be applied to family communication around direction of care and trigger formal brain death evaluation.

